# Clinical Application of 3D Printing Technology in the Production of Canine Full Limb Prosthetics

**DOI:** 10.1155/vmi/9052033

**Published:** 2025-12-15

**Authors:** Shuna Yang, Jianlong Yu, Zhihong Feng, Yufeng Huang, Yuehui Huo, Zhen Zhang, Nan Jiang, Fangzheng Li

**Affiliations:** ^1^ College of Veterinary Medicine, Qingdao Agricultural University, Qingdao, Shandong, China, qau.edu.cn; ^2^ Qingdao Jiazhi Biotechnology Co., Ltd, Qingdao, Shandong, China; ^3^ Qingdao Huichong Pet Hospital Co., Ltd, Qingdao, Shandong, China

**Keywords:** 3D modeling, 3D printing technology, canine, full limb prosthesis

## Abstract

3D printing technology offers innovative and precise solutions for the fabrication of prosthetic devices for pets, leveraging its capabilities in personalized customization, swift response to production demands, and economic viability. This study explores the potential of 3D printing technology in creating customized pet prosthetics, offering an innovative solution for pets experiencing limb loss. A Chihuahua with severe left‐front limb loss was selected as the research subject. After taking precise measurements of its body dimensions, a 3D model of the prosthetic limb was created using 3D Max software, and a full‐limb prosthetic was printed using polylactic acid (PLA) as the material. The prosthetic was then fitted to the canine’s body, and its efficacy was evaluated in detail. The evaluation of the efficacy of the prosthesis used in this study mainly involves observing its adaptability and comfort, functional recovery, durability, and economy. The results indicate that the prosthetic model, designed based on key body size data such as chest circumference, not only conformed to the canine’s physiological characteristics in terms of structure but also effectively supported its body weight, facilitating a recovery to near‐normal ambulatory and locomotor functions. This study demonstrates the feasibility and effectiveness of 3D printing technology in pet prosthetics, providing valuable technical insights for similar clinical cases. In summary, 3D printing technology has shown significant potential in developing customized pet prosthetics. Its personalized design approach can substantially enhance the mobility of injured canines, improve their quality of life, and provide an innovative and efficient solution in the pet medical field.

## 1. Introduction

Pets play an indispensable role in human life, providing not only companionship and comfort but also essential emotional support. The presence of pets enables owners to experience familial warmth, serving as a significant source of solace and joy. Upon returning home after a long workday, the enthusiastic greeting from their pets can swiftly alleviate fatigue and stress [1]. As the pet population continues to grow, the profound emotional impact of pet accidents on owners, as well as the long‐term consequences, has garnered increased attention. When pets face misfortune, the suffering endured by their owners often parallels the grief experienced when a loved one is injured. In the context of trauma treatment for pets, high medical costs frequently compel some owners to make difficult choices. For example, when a dog suffers a severe injury, such as a leg amputation due to an accident, it requires not only trauma treatment but also prosthetics to facilitate recovery. However, the financial burden associated with prosthetics leads many owners to forgo these options, exacerbating their emotional distress. For pets, disabilities signify not just a loss or impairment of physical function but also a significant decline in both quality of life and mobility.

Over the past 15 years, significant advancements have occurred in the design and application of animal orthotics and prosthetics, particularly for dogs and cats [2]. Prosthetics are medical devices that attach to the body; for amputated dogs, they not only replace part or all of a limb but also play a crucial role in weight‐bearing during standing and walking [3]. Essentially, a prosthetic device is affixed to an incomplete limb to restore mobility, while an orthotic is an external device used to support or protect an injured body part. Both prosthetics and orthotics are indispensable components of the rehabilitation process for companion animals [4]. With continuous technological advancements, prosthetic manufacturing techniques have evolved from simple to complex and from basic to advanced. To enhance the practical performance of prosthetics, substantial investments have been made in advanced technologies and materials to create superior devices. While the application of these technologies undoubtedly improves prosthetic performance, it has also contributed to a continuous increase in costs.

3D printing technology, also referred to as additive manufacturing (AM), is a process that fabricates three‐dimensional objects by layering materials based on digital model files. This technique is increasingly applied in the veterinary field due to its ease of use, versatility, and the range of available materials, as well as its relatively low costs, making it an ideal approach for advancements in biomedical solutions [5, 6]. Furthermore, 3D printing is recognized for its high degree of customization and design flexibility, enabling the production of prosthetics tailored to individual needs and assisting in the repair of damaged tissues [5, 7, 8]. Fused deposition modeling (FDM) technology, the most affordable branch of AM [9], has significantly lowered the manufacturing costs of custom prosthetics. Compared to traditional prosthetic manufacturing methods, 3D‐printed prosthetics demonstrate significant advantages, including high customization and fit, reduced manufacturing costs, and enhanced durability. However, 3D‐printed prosthetics also face some limitations, such as challenges in material stability and the high demand for specialized skills.

3D printing technology has made significant progress in addressing the economic and emotional challenges faced by pet owners. 3D printing technology enables more pet owners to afford high‐quality prosthetics by reducing production costs. This technology not only improves the quality of life for pets but also provides emotional comfort for pet owners, allowing them to see injured pets regain mobility. With the continuous advancement and popularization of technology, the application prospects of 3D printed prosthetics in the field of pet medicine are broad, which is expected to further reduce the economic burden of pet owners and meet their expectations for pet health and happiness.

Before designing prosthetic limbs for animals, a comprehensive physical assessment is essential to determine the optimal assembly scheme. Transitioning from quadrupedal to tripod locomotion involves alterations, such as shifts in the center of gravity, reassignment of weight distribution, adjustments in the range of motion of joints, variations in stride, changes in standing duration, and increased load on the remaining limbs [10, 11]. Canines typically support approximately 60% of their body weight with their forelimbs, and the loss of one forelimb can significantly affect their lumbar region [12, 13]. This necessitates that, in the design of prosthetics, we consider not only their supportive role but also how to minimize the stress on the animal’s other body parts.

As technology advances, 3D scanning offers innovative solutions for designing and manufacturing prosthetic limbs for animals [14]. This technology can efficiently capture the shape data of an animal’s limb and reconstruct an accurate 3D model, significantly reducing the time required to design prosthetic devices in the medical field, particularly for experienced clinical practitioners [15]. Mendaza‐Decal has documented the process of creating animal orthoses and prosthetics using 3D scanning and FDM technology; this innovative approach not only expedites production but also enhances the fit and comfort of the prosthetics. With this technology, we can provide more personalized and precise prosthetics for animals, aiding them in adapting to and resuming their daily activities [16]. Prior research has primarily focused on dogs with intact elbow joints, which can be directly fitted with prosthetics. However, there has been limited in‐depth research on prosthetic solutions for cases of significant limb loss, such as disabled dogs with only the shoulder joint remaining.

In this research, the computer‐aided design and 3D printing technology were employed using polylactic acid (PLA) material to custom‐tailor a prosthetic for a dog that had suffered severe loss of its left forelimb. This intervention not only reestablished the canine’s fundamental ambulatory function but also substantially improved its comfort and autonomy in everyday activities. This research indicates the significant potential and efficacy of 3D printing technology in the realm of animal prosthetic customization, setting the groundwork for its broader adoption and advancement within veterinary medicine. Therefore, the purpose of this study is to explore the technical process of 3D printing technology in the field of dog personalized prosthesis manufacturing, verify its feasibility, and provide reference for similar clinical cases.

## 2. Materials and Methods

### 2.1. Case Selection

A ten‐year‐old female Chihuahua, weighing 3.5 kg, has a well‐proportioned body and is free from chronic diseases such as heart disease, diabetes, or arthritis, with a normal immune system. It sustained a severe crushing injury to its left forelimb in a car accident in October 2022. One month postsurgery, the microcirculatory system in the anterior part of the affected limb was successfully reestablished, providing hope for recovery. During the second surgery, an attempt was made to temporarily bury the affected limb within the abdomen using a skin graft to promote healing. Unfortunately, this approach proved unsuccessful due to insufficient burial time and physiological effects associated with age. The third surgery entailed amputation of the affected limb at the forearm level; however, postoperative recovery was challenging. The wound frequently came into contact with the ground, lacking adequate muscle tissue for protection, which impeded healing and resulted in eventual necrosis. In the fourth surgery, a difficult decision was made to amputate at the midpoint of the arm bone, leaving only a short 2 cm stump.

### 2.2. Data Measurement

To develop a preliminary model diagram for the dog’s prosthesis, measurements were taken of the dog’s neck circumference, the length and leg circumference of the intact right forelimb, chest circumference, abdominal circumference, the distance from the midline of the abdomen to the midline of the back, and the length of the stump of the left forelimb. Use a soft leather tape measure to measure neck and chest circumference, fitting the body curve to avoid discomfort. Use a ruler to measure the length of limbs. Use a tape measure to measure abdominal circumference and a ruler to measure the distance from the midline of the abdomen to the midline of the back and the length of the residual limb. These measurement methods not only ensure the accuracy of data but also minimize discomfort to pets during the measurement process.

### 2.3. Sketch Design and 3D Model Construction of Prosthetic

Based on the condition of the left forelimb stump and the fixation method, a planar sketch of the prosthetic was created referencing the right forelimb. Subsequently, using the measurement data, a 3D model was constructed in 3D Max software. First, import the planar sketch of dog prosthesis into the 3D Max scene and adjust its size according to the measured dimensions. Use the sketch as a reference to create the basic models of the receiving cavity and the baseboard through lofting modeling techniques. Construct the foundational model of the supporting rod using a cylinder as the base. Next, refine the basic model using the Edit Polygons function, ensuring that it appears smooth and natural while conforming to the dog’s body curve. Create holes in the receiving cavity by employing Boolean operations to reduce the weight of the prosthesis. Finally, apply the TurboSmooth modifier in 3D Max to enhance the model’s surface. All components were adjusted to ensure a proper fit at the joints, resulting in a complete 3D model of the prosthetic.

### 2.4. 3D Printing of Prosthetics

The individual components of the 3D model of the prosthetic were exported in STL format (.stl) and imported sequentially into the 3D printing software, where they were appropriately positioned. The models were printed using the Einstart‐L desktop 3D printer from Shenzhen Anycubic Technology Co. Ltd. (Shenzhen, Guangdong, China), utilizing PLA material also provided by the company. Set the printing parameters of the model, including setting the nozzle temperature to 205°C and setting the layer height to 0.2 mm, printing distance to 80 mm/s, and filling spacing to 5 mm. After printing, the support structures were removed to obtain the individual printed components, which were subsequently assembled to construct the complete prosthetic model.

### 2.5. Fitting Prosthetics

After equipping the dog with the prosthetic, the duration of usage should be gradually increased. Initially, during the initial adaptation phase, the prosthetic should be worn for 30 min per day, then increased to 1 h per day during the intermediate adaptation phase, followed by 2 h per day in the enhanced adaptation phase, and finally worn for the entire day for long‐term adaptation. Throughout this process, the dog’s reactions to the prosthetic should be closely monitored for any signs of discomfort. Afterward, observe the changes in dogs at different times after installing prosthetics on a weekly basis. Timely adjustments should be made to the prosthetic as needed to ensure an optimal fit.

### 2.6. Ethical Approval

Ethical approval was obtained from the Institutional Review Board of the Qingdao Agricultural University. The study methods were performed in accordance with approved guidelines.

## 3. Results

### 3.1. Information of the Affected Dog

The dog’s neck circumference is 24.5 cm, the length of the intact right front leg is 20.5 cm, the leg circumference is 10 cm, the chest circumference is 38 cm, the girth is 43.5 cm, the abdominal circumference is 41.5 cm, and the distance from the midline of the abdomen to the midline of the back is 17.5 cm. The distal end of the dog’s left front leg retains approximately 2 cm of the humerus (Figure 1(a)). The X‐ray image clearly shows the state of the dog’s left front limb stump (Figure 1(b)).

Figure 1Basic information of the affected dog. (a) Left lateral view of the affected limb in the dog. (b) Radiograph of the affected limb in the dog.

**Figure:**
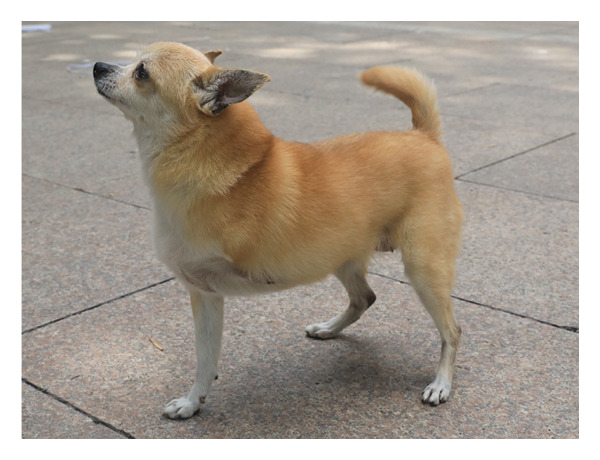
(a)

**Figure:**
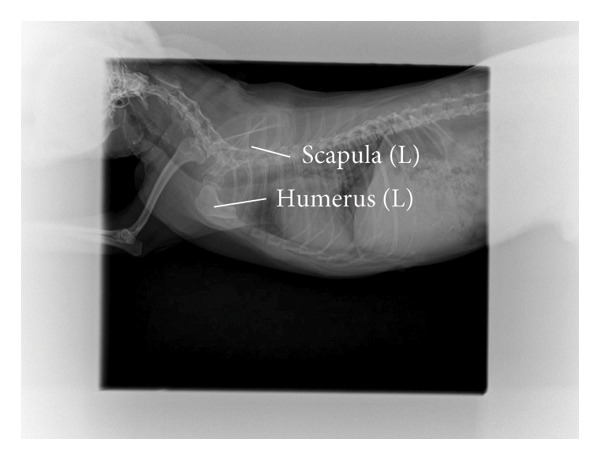
(b)

### 3.2. Sketch Design and 3D Model Construction of Prosthetic

Due to the severe loss of the dog’s forelimb, a full limb prosthetic (Figure 2(a)) was designed, comprising four parts: the thoracic socket, the base, the support rod, and the footplate, with each part meticulously crafted to meet the dog’s physiological requirements (Figure 2(b)). The socket encloses the dog’s chest, anterior abdomen, and abdomen, featuring an opening on the lower right side for the right forelimb to protrude and a circular arc recess on the left to accommodate the amputated limb. The recess has a support structure at the bottom to maintain the overall stability of the receiving socket and to facilitate secure attachment to the base. The upper part of the receiving socket has three openings for Velcro straps to serve as a fixation function. The receiving socket is designed with a perforated pattern to enhance breathability and reduce weight, making it suitable for extended wear. The base beneath the receiving socket has holes for connecting to the support rod, with the rod below being cylindrical, and the footplate at the bottom also featuring a perforated design (Figure 2(c)).

Figure 2Design of canine prosthetic. (a) Design ideas for canine prosthetics. (b) Draft of canine prosthetics. (c) 3D model of canine prosthetic limb.

**Figure:**
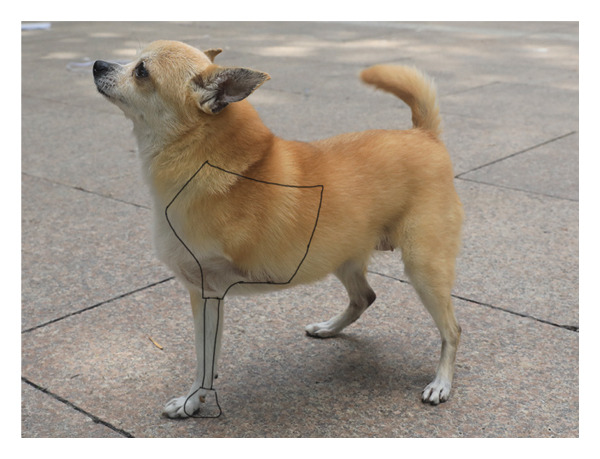
(a)

**Figure:**
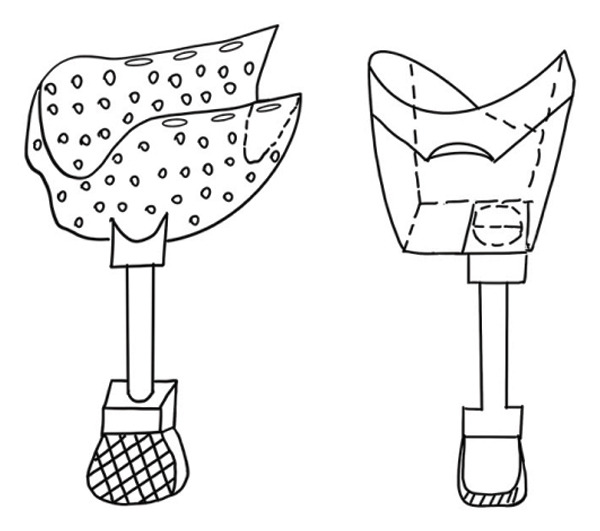
(b)

**Figure:**
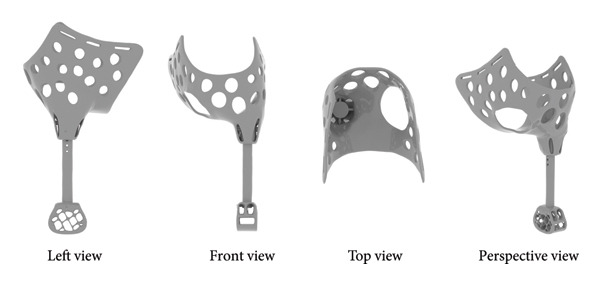
(c)

### 3.3. 3D Printing of Prosthetics

The 3D model completed in 3D Max is exported as a ^∗^.stl format file, then imported into the 3D printer’s slicing software, 3Dstar, to adjust the position for slicing, obtaining the slicing path for the prosthetic (Figure 3(a)). The printing of the model took 24.26 h, using 64.4 m of PLA filament, weighing 166 g, and the material cost is less than ¥100. After the printing is completed, the individual components are separated, and once the support structures are removed, the individual component models can be obtained. These parts are then connected to assemble the complete prosthetic model (Figure 3(b)).

Figure 33D printing of prosthetics. (a) 3D printing settings for prosthetic models. (b) The assembled prosthetic model.

**Figure:**
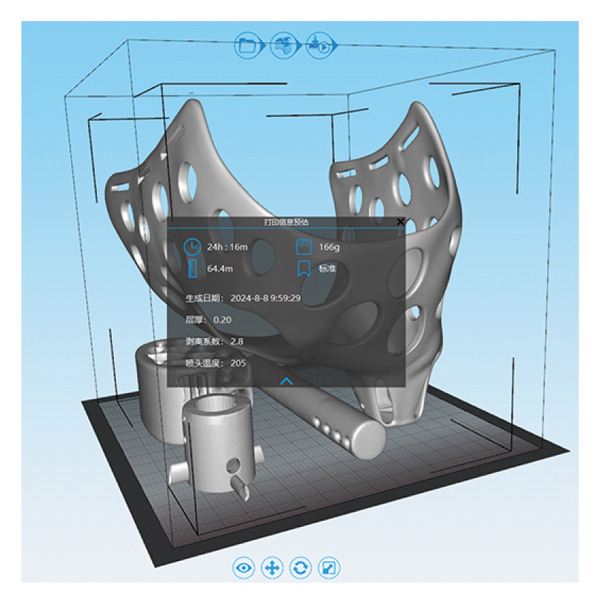
(a)

**Figure:**
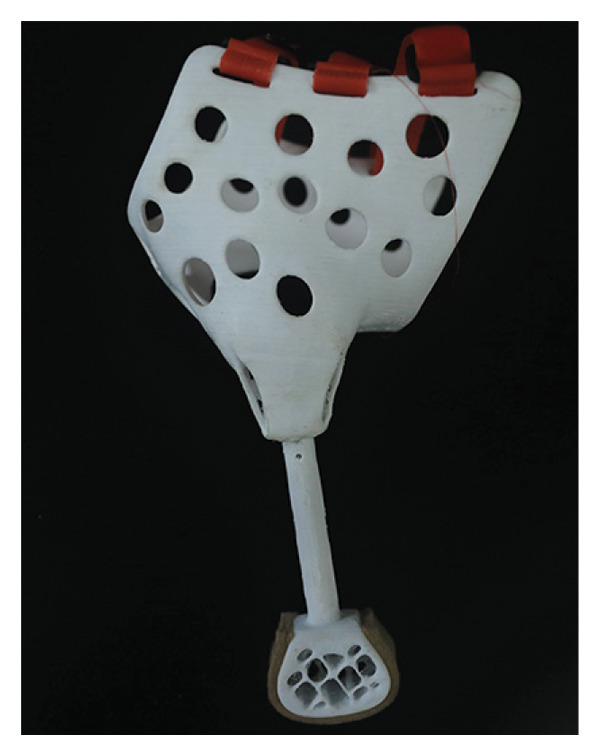
(b)

### 3.4. Fitting Prosthetics

Attach and fit the prosthetic to the dog using Velcro straps and observe its adaptability and comfort level. In the early stages of adaptation, the dog exhibited minor discomfort, such as limping, but these behaviors notably diminished within a few days. With the extension of wearing time, the dog’s gait gradually stabilized, and its range of motion correspondingly increased, particularly during the mid‐adaptation phase. By the end of the experiment, the dog was capable of wearing the prosthetic throughout the day, after wearing prosthetic limbs, the dog’s gait is normal, and there is no redness, swelling, or ulceration in the contact area of the residual limb, and it is lively and active, demonstrating good adaptability and comfort (Figure 4).

Figure 4Installation of prosthetic limbs for the affected dog. (a) Left lateral view of the dog with a prosthetic limb. (b) Frontal view of the dog with a prosthetic limb. (c) Right lateral view of the dog with a prosthetic limb.

**Figure:**
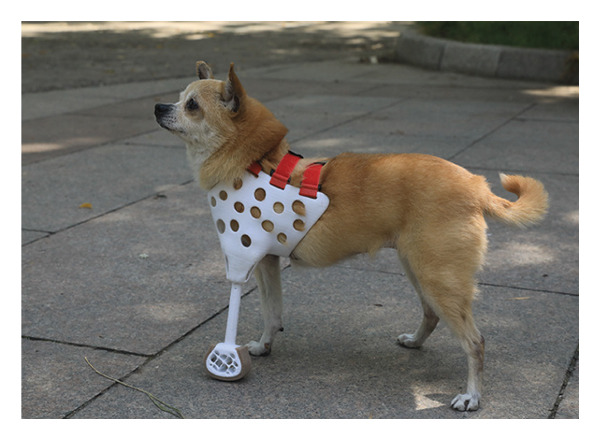
(a)

**Figure:**
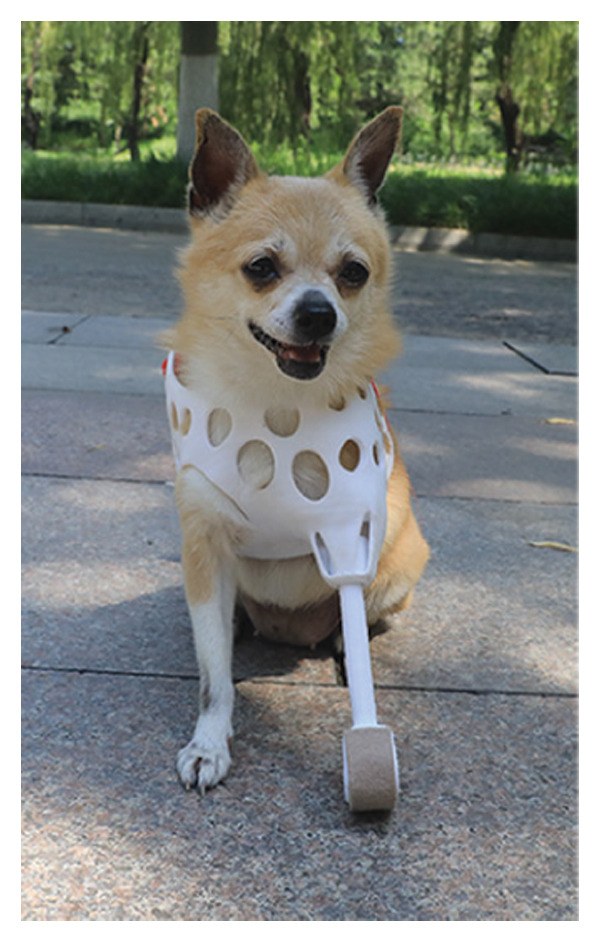
(b)

**Figure:**
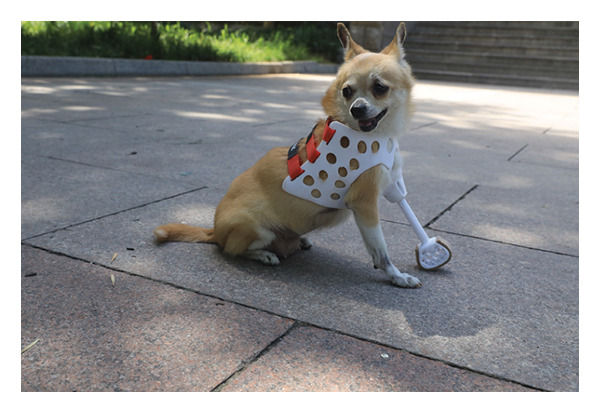
(c)

## 4. Discussion

The application of 3D printing technology in the medical field is developing rapidly and shows significant potential for growth. Currently, this technology is capable of custom‐making prosthetics for disabled pets, significantly enhancing their quality of life and freedom of movement [17]. This research focused on a dog with severe forelimb damage and successfully designed and created a complete limb prosthetic using 3D printing technology, providing stable support for the dog’s daily activities. Moreover, 3D‐printed prosthetics, by reducing manufacturing costs and enhancing customization and precision, offer pet owners a more affordable and efficient prosthetic solution. For example, the prosthetic limb in this study is mainly designed with a labor cost, while the material cost is less than ¥100. The application of 3D printing technology in the manufacturing of animal prosthetics has demonstrated significant potential. Its ability to provide personalized customization can address the unique needs of various animals, including dogs, cats, and other wildlife. With the heightened focus on animal welfare, the demand for customized prosthetics within the veterinary field is rapidly increasing. Numerous veterinary clinics and animal protection organizations have begun to explore the use of 3D printing technology to aid injured or disabled animals. This trend suggests that expanding the production of 3D‐printed prosthetics not only meets market demand but also enables assistance for a broader range of animals.

The personalized design approach employed in this study ensures a seamless fit between the prosthetic and the dog’s body by precisely measuring the residual limb and body dimensions. This design concept is innovative within the field of prosthetics, as it considers not only the functionality of the prosthetic but also the dog’s comfort and adaptability. The design and fabrication of full limb prosthetics for pets present unique challenges; this study offers a novel solution for pets with severe limb loss, marking a significant advancement in the personalized customization of pet prosthetics. The rationale behind the prosthetic structure is essential for its functionality and comfort [16]. In this case, due to the dog’s short residual limb, traditional methods were inadequate for securely attaching the prosthetic. Therefore, we initially designed a thoracic receiving socket that could be fitted to the dog’s body, serving as the key structure for prosthetic fixation. It needed to fit snugly to provide adequate support while considering the dog’s breathing and movement to prevent any compression or restriction. Additionally, the thoracic receiving socket functions to connect and fix the prosthetic securely to its base, achieving an effective link between the prosthetic and the dog’s body. The hollow design of the receiving socket not only reduces the weight of the prosthetic but also enhances breathability. This design improves the comfort of the dog wearing the prosthetic and minimizes its impact on the dog’s daily activities. Our aim is to significantly enhance the dog’s mobility and quality of life, enabling it to resume normal activities and live as fully as possible.

The application of 3D printing technology in this study highlights the significant potential of contemporary manufacturing techniques in prosthetic fabrication. The three‐dimensional models created using 3D Max software not only accurately reflect the geometric form of the prosthetic but also facilitate easy resizing to accommodate animals of various sizes [18]. Moreover, 3D printing has substantially reduced production time, lowered costs, and enhanced precision and complexity. It allows for rapid iteration and customization to meet the specific needs of individual dogs, offering a level of flexibility that traditional manufacturing methods often lack. Although 3D scanning can quickly capture the morphology of a patient’s residual limb, thereby aiding in prosthetic design [16], it was inconclusive in this instance due to the Chihuahua’s long and fluffy fur. The models produced from 3D scanning included the fur length, causing them to misrepresent the true body contour, which would result in poorly fitting prosthetics. Consequently, this study employed manual measurement techniques, appropriately scaling based on the initial Chihuahua model to create a prosthetic that better conforms to the patient’s body shape.

In evaluating the prosthetic’s adaptability and comfort, this study utilized detailed observation methods and extensive tracking [16]. During the initial adaptation phase, the dog’s mild discomfort, such as limping and frequent licking of the prosthetic area, was typical for the adjustment process. However, as the dog acclimated to the prosthetic, these behaviors notably diminished, indicating the design’s effectiveness. During the mid‐adaptation phase, the dog’s improved gait stability and increased range of motion further validated the design rationale. By the experiment’s final stage, the dog could wear the prosthetic throughout the day, exhibiting good adaptability and comfort in line with prior research on prosthetic adaptability assessment standards [19].

While this study has achieved notable outcomes in prosthetic design and production, it is not without limitations. The small sample size, focusing on a single canine subject, restricts the generalizability of the results. Despite the significant potential of 3D printing technology in the production of dog prostheses, several limitations persist. Firstly, the accuracy and reliability of current 3D printers require improvement, which may result in improper fitting of the prosthesis to the dog’s body and potential printing failures. Secondly, existing 3D printing materials often exhibit deficiencies in biocompatibility, strength, and wear resistance. Furthermore, the long‐term efficacy and durability of the prosthetic are yet to be thoroughly evaluated, which is critical for assessing its practical value. Future studies should aim to increase the sample size to include a diverse range of canine breeds, ages, and weights, thereby enhancing the study’s representativeness and reliability. Concurrently, long‐term monitoring of the prosthetics should be conducted to evaluate their endurance and adaptability over time. Additionally, future research could explore innovative materials and manufacturing technologies, such as smart materials and advanced fabrication techniques, to improve the performance and comfort of prosthetics.

Selecting wear‐resistant and biocompatible materials is fundamental to ensuring the durability of prosthetics. In future research, alternative materials such as biodegradable or more durable plastics will be used in 3D printing for the production of prostheses to adapt to different application scenarios. Enhancing the performance and lifespan of prosthetics can be achieved through optimized design and manufacturing processes. By providing comprehensive maintenance guidelines and technical support to extend the lifespan of prosthetics, a more in‐depth analysis of the long‐term durability and maintenance of 3D‐printed prosthetics can be conducted, thereby promoting their practical application.

This study successfully designed and produced a full limb prosthetic using 3D printing technology. This achievement not only represents a technical innovation but also demonstrates significant adaptability and comfort in practical applications. The personalized prosthetic design approach serves as an effective aid for dogs with severe forelimb defects, thereby enhancing their quality of life. Despite limitations in sample size and the assessment of long‐term effects, the findings offer valuable experience and insights for advancing the pet prosthetic sector. From a more forward‐looking perspective, the future research directions of 3D printing technology in prosthetic manufacturing can achieve more natural motion control and tactile feedback, while producing prosthetics that are lighter, more durable, and functionally complex. Future research should refine prosthetic designs based on existing foundations, investigate new materials and technologies, and broaden the research’s scope and depth to provide more comprehensive solutions that assist a larger number of pets in need.

## Conflicts of Interest

The authors declare no conflicts of interest.

## Author Contributions

Conceptualization: Fangzheng Li and Shuna Yang; data curation: Jianlong Yu, Zhihong Feng, and Zhen Zhang; funding acquisition: Fangzheng Li and Nan Jiang; methodology: Fangzheng Li, Yufeng Huang, and Yuehui Huo; visualization: Zhen Zhang and Yufeng Huang; writing–original draft: Nan Jiang and Shuna Yang; and writing–review and editing: Fangzheng Li. Shuna Yang and Jianlong Yu equally contributed to this work.

## Funding

This research was supported by the Project of Qingdao Emerging Industry Cultivation Plan (23‐1‐4‐xxgg‐21‐nsh) and National Natural Science Foundation of China (32202756).

## Data Availability

We declare, to who may be interested, that access to all of the data of this manuscript can be obtained from the corresponding author, Fangzheng Li, by email fzli78@163.com.
